# Twenty-Four Week, Randomized, Double-Blind, Placebo-Controlled Trial of Metformin for Antipsychotic-Induced Weight Gain in Patients with First-Episode Psychosis: A Pilot Study

**DOI:** 10.3390/ijerph19010137

**Published:** 2021-12-23

**Authors:** Charmaine Tang, Yi Chian Chua, Edimansyah Abdin, Mythily Subramaniam, Swapna Verma

**Affiliations:** 1 Department of Psychosis, Institute of Mental Health, Singapore 539747, Singapore; yi_chian_chua@imh.com.sg; 2 Research Division, Institute of Mental Health, Singapore 539747, Singapore; Edimansyah_Abdin@imh.com.sg (E.A.); Mythily@imh.com.sg (M.S.); 3 Medical Board, Institute of Mental Health, Singapore 539747, Singapore; Swapna_Verma@imh.com.sg; 4 MD Programme Department, Duke-NUS Medical School, Singapore 169857, Singapore

**Keywords:** metformin, antipsychotic-induced weight gain, first-episode psychosis

## Abstract

Excessive weight gain and cardiometabolic dysfunction are common and clinically relevant side effects of antipsychotic medications. In this pilot study, we aimed to establish the feasibility of using metformin and its effectiveness in managing antipsychotic-induced weight gain in patients with first-episode psychosis (FEP) on follow-up with the Singapore Early Psychosis Intervention Programme in a 24-week, randomized, double-blind, placebo-controlled trial, to ascertain the effects of metformin discontinuation on body weight and evaluate the safety and tolerability of metformin. Participants between the ages of 16 and 40 with FEP assessed as clinically stable and who had gained ≥5% of their pre-drug weight after initiation of the antipsychotic treatment were recruited from outpatient clinics between April 2015 and April 2018. Seventeen participants met all the inclusion criteria and were randomized to receive metformin (n = 8) or the placebo (n = 9) at Week 0, with follow up assessments at Weeks 3, 6, 12, 24, and 36. Metformin was generally well-tolerated. Participants in the metformin arm were able to control their weight better than participants receiving the placebo, an effect that did not persist after discontinuation. Our results support the use of metformin as a safe and tolerable weight control measure in a typical outpatient sample of young people with FEP.

## 1. Introduction

It is well established that early and effective treatment in patients with first-episode psychosis (FEP) improves treatment response and functional outcomes [1]. Antipsychotic medications remain the gold standard in managing such patients, with studies revealing better clinical response to antipsychotics in patients with FEP compared to patients with chronic schizophrenia [2]. However, the risk–benefit ratio of antipsychotics is negated by their side effects. Excessive weight gain and cardiometabolic dysfunction are common and clinically relevant side effects of antipsychotic medications [3,4,5]. They not only influence adherence [6], but are also associated with substantial morbidity and mortality. Patients with schizophrenia are twice as likely to have metabolic risk factors, and die approximately 20 years earlier than the general population, with coronary heart disease accounting for at least 50% of this excess mortality [7,8]. Although the reasons for increased cardiovascular disease in schizophrenia are multifactorial, adverse metabolic side effects of antipsychotic medications play an important role. In a Singaporean study examining the effects of antipsychotic treatment on weight gain and metabolic abnormalities in patients with FEP, the authors found significant increases in body mass index (BMI), serum levels of triglyceride, low-density lipoprotein (LDL), and total cholesterol (TChol) from baseline to 6 months, with 65% of patients having clinically significant weight gain (i.e., ≥7% increase from baseline) [9]. More convincingly, when the authors set out to identify the prevalence of cardiovascular risk factors in drug-naïve patients with FEP compared to healthy controls matched for age, gender, and ethnicity, they found that the mean baseline weight, BMI, TChol, and LDL were significantly higher in controls compared to patients [10]. These findings suggest that weight gain and metabolic dysfunction in patients with FEP are associated effects of antipsychotic medications.

Patients with FEP are a particularly vulnerable group given that these side effects carry the serious health risks of metabolic syndrome in young individuals who may potentially need long-term, if not lifelong antipsychotic treatment. The dual stigma of not only having a mental illness but also being overweight is likely to result in low self-esteem and further lead to social discrimination and isolation in these young people [11]. Given the magnitude of the problem, the progress in improving antipsychotic-induced weight gain (AIWG) and metabolic dysfunction has been disappointing [12]. Recommendations to implement lifestyle changes and to switch the patient from an antipsychotic with high metabolic liability to one with a lower liability are more often than not challenging. Switching antipsychotics in patients stabilized on a given drug may result in relapses [13]. Attempts at lifestyle changes are often not successful due to antipsychotic-induced increased appetite, illness-related factors, and lack of patient motivation [14,15].

Metformin is an oral biguanide antidiabetic agent that is widely prescribed for the treatment of non-insulin-dependent type 2 diabetes mellitus (DM). It is regarded as a weight-neutral agent in contrast to other oral antidiabetic agents, and is postulated to act by reducing hepatic gluconeogenesis, enhancing insulin sensitivity by increasing peripheral glucose uptake and utilization and reducing glucose absorption from the gastrointestinal tract [16,17]. It has also been used in non-diabetic non-psychiatric populations for weight reduction and prevention of type 2 DM in high-risk cohorts with modest success [18]. Contributing mechanisms for weight loss are thought to include appetite suppression and slowing of gastric emptying related to stimulation of glucagon-like peptide-1 secretion [19,20]. In addition, it is well-tolerated and has a well-established safety profile in both adults and youths. Lactic acidosis is extremely rare, and the most common side-effects are nausea, vomiting, and diarrhea [21]. Accumulating evidence suggests that metformin augmentation of antipsychotics is an evidence-based option to reduce antipsychotic-induced cardiometabolic effects [22,23,24,25].

In this pilot study, we aimed to firstly establish the feasibility of using metformin and evaluate its effectiveness in managing AIWG in patients with FEP on follow-up with the Singapore Early Psychosis Intervention Programme (EPIP) in a 24-week, randomized, double-blind, placebo-controlled trial; secondly, assess body weight at 12 weeks after completion of the trial, so as to ascertain the effects of metformin discontinuation on body weight; and thirdly, evaluate the safety and tolerability of metformin. We hypothesized that metformin will produce greater weight loss and have beneficial effects on metabolic profiles compared to the placebo. We also hypothesized that weight loss in patients may not be sustained after discontinuation of metformin.

## 2. Materials and Methods

### 2.1. Participants

Participants between the ages of 16 and 40 with first-episode psychotic disorder, whereby psychotic disorder was defined as meeting the DSM-IV criteria for schizophrenia, schizophreniform disorder, schizoaffective disorder, delusional disorder, brief psychotic disorder, or mood disorders with psychotic features, were recruited from EPIP outpatient clinics between April 2015 and April 2018. EPIP is a nationwide program in Singapore catering specifically to patients experiencing their first episode of psychosis, and providing comprehensive multidisciplinary team management with psycho-pharmacological treatment emphasizing the use of antipsychotic monotherapy. Diagnoses were determined by the Structured Clinical Interview for DSM-IV Axis I Disorders, Clinician Version (SCID-CV) [26]. To be eligible for the study, patients had to be clinically stable as assessed by their treating psychiatrist and having a Clinical Global Impressions—Severity Scale (CGI-S) [27] score of ≤3; had to have gained ≥5% of their pre-drug weight after initiation of antipsychotic treatment (≥5% increase from baseline weight is clinically significant weight gain in accordance with the US Food and Drug Administration guidelines); and had to be able to provide informed consent where their competence to consent was determined by their treating psychiatrist. Female participants of childbearing potential had to have a negative pregnancy test at screening and pre-dose, and had to be willing to practice adequate methods of contraception during the study. Patients were excluded from the study if there was evidence from case records or current screening blood tests of thyroid, liver or renal dysfunction, cardiovascular disease, or DM; current or previous treatment with metformin or other antidiabetic agents; known allergy to metformin; if they were pregnant or lactating; if they had any major and unstable medical or neurological illness; if they had a baseline BMI < 18.5 kg/m^2^ (the cut-off point for underweight adults as per World Health Organization (WHO) guidelines); if they had intellectual disability; if they had current alcohol and/or substance abuse or dependence; or if they had used any medication for weight loss within the preceding month prior to study entry. Enrolled participants were subjected to early termination should incidental findings (such as abnormal laboratory findings) or failure to comply with study procedure occur during the trial.

### 2.2. Study Design

The study protocol was approved by the National Healthcare Group Domain Specific Review Board (DSRB Ref. No.: 2013/01037) and registered with the Health Sciences Authority Clinical Trials Registry (Protocol No.: 2013-01037). This was a 24-week, randomized, double-blind, placebo-controlled trial comparing metformin and placebo for AIWG in patients with FEP. There was an additional discontinuation phase whereby participants were assessed 12 weeks after completion of the trial (i.e., Week 36) so as to ascertain the effects of metformin discontinuation on body weight. Participants were identified and screened for eligibility during the screening phase based on protocol inclusion/exclusion criteria within 1 week prior to enrolment into the study. Participants who were enrolled into the study were randomly assigned in a 1:1 ratio to receive either the metformin or placebo arm at Week 0, with follow up assessments at Weeks 3, 6, 12, 24, and 36. Randomization was carried out using a computer-generated table to assign eligible patients to one of the 2 treatment groups in blocks of 4 to ensure equal numbers of participants in the 2 groups. To ensure the concealment of the randomization, metformin and the identical-appearing placebo were provided by coded and opaque containers. Patients, caregivers, investigators, and research assistants were blinded to the treatments. The randomization assignments were decoded only at completion of the trial or early termination.

### 2.3. Procedures

A comprehensive study schedule listing all the procedures and assessments carried out at each study visit is presented in Table 1. During the screening phase, all participants underwent an informed consent process, during which voluntary participation was requested and written informed consent was obtained. For participants under 21 years of age, consent was obtained from their legally acceptable representative. The sociodemographic and clinical characteristics collected are described in Table 2, as well as anthropometric measures and vital signs listed in Table 3. In addition, participants underwent a physical examination of their head and neck, heart, lungs, abdomen, limbs, and neurological systems by their treating psychiatrist, a 12-lead electrocardiogram conducted in-house, blood investigations, and for female participants of childbearing potential, an additional serum pregnancy test. Blood investigations included serum creatinine, estimated glomerular filtration rate (eGFR), serum insulin, lactic acid, aspartate transaminase (AST), alanine transaminase (ALT), haemoglobin A1c (HbA1c), fasting glucose, thyroid stimulating hormone (TSH), free thyroxin (fT4), TChol, high-density lipoprotein (HDL), LDL, and triglycerides. Blood samples were collected on-site by clinical staff nurses after participants fasted for at least eight hours and sent to a partner laboratory for analyses. Upon receipt of the laboratory results, the treating clinician would note any abnormal findings, and should the participant meet all the inclusion and none of the exclusion criteria, formally enroll the participant into the study.

At Week 0, participants were given a 500 mg metformin tablet or placebo once nightly. Telephone sessions with the participants were conducted by the study administrators at Weeks 1 and 2 for concomitant medication review and adverse event monitoring, as well as to remind participants about appropriate medication usage and adherence. If the study drug was assessed to be well tolerated, this was increased to one tablet twice daily from Week 1 onward, followed by one tablet in the morning and 2 tablets at night from Week 2 onward. 

For the remaining visits, a drug accountability and adherence (defined as ≥80% compliance) check was done for the remaining study medication brought back by participants at each visit, and the metformin or placebo was dispensed accordingly. Other study procedures and assessments were also repeated according to the study schedule in Table 1.

### 2.4. Assessments

At Weeks 0, 24, and 36, participants were rated by their treating clinicians on the Brief Psychiatric Rating Scale (BPRS) [28] and the Global Assessment of Functioning (GAF) [29]. Out of the clinicians in EPIP, five were treating participants enrolled in the study and were involved in conducting the relevant assessments. The 18-item version of the BPRS was used, in which the participants’ symptoms and signs (e.g., somatic concern, anxiety) were rated from 1 (Not present) to 7 (Extremely severe). For the GAF scale, clinicians rated participants in terms of psychological, social, and occupational functioning on a hypothetical mental health–illness continuum ranging from 0 to 100.

At the same time points, participants also completed a battery of questionnaires, including the Patient Health Questionnaire (PHQ-9) [30], Dietary Practices Questionnaire (DPQ) [31], and International Physical Activity Questionnaire (IPAQ) [32]. The PHQ-9 is a 9-item self-reported questionnaire with an additional functional health question, where participants report how often over the last two weeks, on a scale of 0 (Not at all) to 3 (Nearly every day), they felt bothered by various problems (e.g., “Little interest or pleasure in doing things”). The DPQ was created by the Research & Evaluation Department of the Health Promotion Board, Singapore, and consists of 31 items on dietary practices (e.g., “Have you ever been on a diet to lose weight?”) where participants answer with an option (e.g., “Have dieted occasionally, in the past”, “Have dieted frequently, in the past”, “Continually dieting to lose weight”, or “Never dieted”). The IPAQ is a 27-item self-reported questionnaire, where participants report if they took part in various physical activities over the last seven days and how much time they spent doing those activities, as well as the amount of time they spent sitting a day. The BPRS, GAF, and PHQ-9 have been validated for use in psychosis populations [33,34,35], while the DPQ has not. However, it was deemed suitable for use as a descriptive measure for the participants’ dietary habits as it included items specific to Singapore’s food culture. On the other hand, the IPAQ is one of the most commonly used measures for self-reported physical activity [36], but a paper recently published after the commencement of the current study has recommended caution with its use in FEP populations [37].

### 2.5. Outcomes

In this study, the primary outcome measures were body weight across time and the evaluated safety and tolerability of metformin. All other measures collected served as additional descriptive factors.

### 2.6. Statistical Analysis

Using IBM SPSS 23, the mean and standard deviations were computed for continuous variables, and frequencies and percentages for categorical variables. Chi-square and Wilcoxon two-sample tests were used to examine demographic and clinical differences between the metformin and placebo groups. SAS software was used to conduct a mixed model analysis [38] to compare the weight outcomes between the two groups over the first 24 weeks, and a repeated measures analyses of variance (RMANOVA) to compare differences in weight between the two groups at Week 36, during the discontinuation phase. Statistical significance for this study was established at *p*-value < 0.05.

## 3. Results

### 3.1. Participants

**Figure 1:**
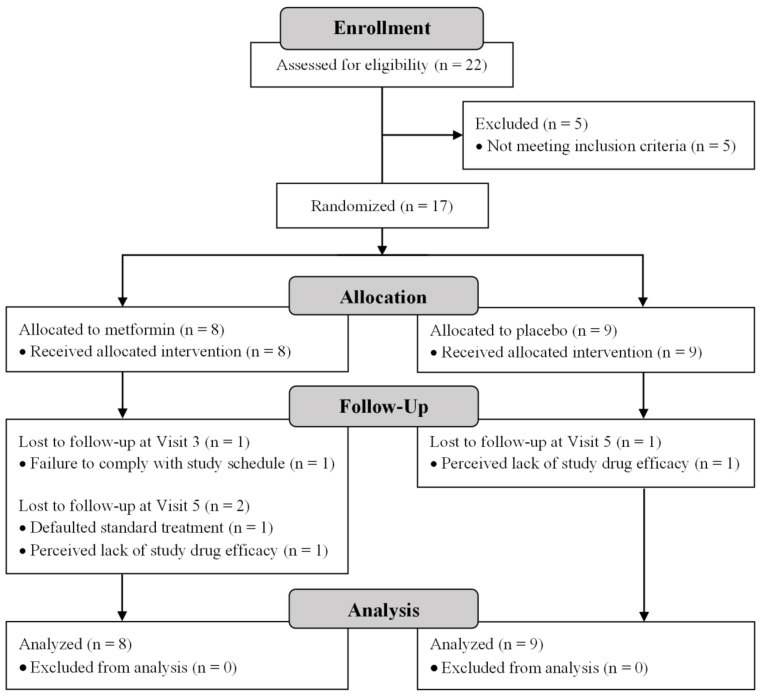
CONSORT diagram of participant flow.

Between April 2015 and April 2018, potential participants were approached at EPIP outpatient clinics. The majority declined to participate, citing reasons such as discomfort with the randomization process (possibly having to spend 24 weeks on a placebo before receiving the appropriate treatment), general reluctance to commit, and unwillingness to fast for eight hours prior for blood tests and then travel down to the study site for clinical measurements. A resulting total of 22 patients consented to the study, seventeen of which met all the inclusion criteria and were randomized to receive either metformin or the placebo. The participant flow is described in Figure 1 according to the Consolidated Standards of Reporting Trials (CONSORT) diagram. Two participants on metformin defaulted treatment and were uncontactable, at Weeks 3 and 12, respectively. Another two participants, one on metformin and one on the placebo, withdrew due to a perceived lack of study drug efficacy at Week 12. Sixteen participants had complete data for up until Week 12, and 13 had complete data up until the end of trial with additional data after discontinuation of the study medication. The baseline demographic and clinical characteristics of the 17 enrolled participants are reported in Table 2, with no statistically significant differences between the metformin and placebo groups other than diastolic blood pressure (*p* = 0.03), suggesting that the randomization process was successful. Information on concurrent medications taken by the participants are also included in Table 2. There were no major changes in the medications taken by the participants throughout the course of the study, even while medication titration continued as part of the participants’ clinical treatment.

### 3.2. Safety and Tolerability

The average dose of metformin taken by participants was 1201.2 mg/day. Metformin was generally well-tolerated and no participants dropped out of the study due to safety and tolerability issues. One participant experienced diarrhea and episodes of nausea and vomiting, which were reported as mild and managed by their family physician. They were also advised to reduce the study drug dosage from one tablet in the morning and two tablets at night to one in the morning and one at night. Other adverse events experienced by other participants included diarrhea (n = 1) and soft stools (n = 1), which were reported as mild and tolerable, and resolved spontaneously. Another participant experienced a psychotic relapse not related to the study protocol and was subsequently admitted during the discontinuation phase. For participants on placebo, adverse events experienced included nausea (n = 1), vomiting (n = 2), diarrhea (n = 1), flatulence (n = 2), indigestion (n = 1), asthenia (n = 1), headache (n = 1), dizziness (n = 1), and light-headedness (n = 1), which were all reported as mild and tolerable. Participants experienced symptoms ranging from very mild to mild, as rated on the BPRS at baseline, except for one participant in the placebo arm who presented with a moderately severe somatic concern. At Weeks 24 and 36, these symptoms were either resolved, or remained at very mild to mild severity. The GAF scores also showed a general upward trend for all over time.

### 3.3. Outcomes

The clinical parameters of participants over time is described in Table 3. Of the five participants on metformin who completed the trial in its entirety, four lost weight at Week 24 compared to Week 0 (average of 1.9 kg), while one gained weight (1.0 kg). Of the eight participants on placebo that completed the trial in its entirety, seven on placebo gained weight at Week 24 (average of 3.8 kg) compared to Week 0, while one on placebo lost weight (1.9 kg). Of the participants that dropped out at Week 12, one on metformin gained weight (3.1 kg) and one lost weight (3.6 kg), while the one on placebo gained weight (9.2 kg).

The results of the mixed model analysis (Table 4) showed that the interaction effect of treatment during the intervention phase (from Week 0 to 24) on weight was found to be statistically significant, which supports a significant difference between the metformin and placebo groups on weight outcomes. During the discontinuation phase, between Weeks 24 and 36, three out of the five participants on metformin gained weight (average of 3.4 kg) while two lost weight (average of 2.3 kg), and seven out of the eight on placebo gained weight (average of 1.8 kg) while one maintained. This brought two of the participants on metformin above (average of 3.0 kg) and three below (2.4 kg) their baseline body weight, and seven of the participants on placebo above (average of 5.4 kg) above their baseline body weight. The RMANOVA (Table 4) conducted found no statistically significant difference between the metformin and placebo groups during the discontinuation phase (from Week 24 to 36).

No major changes were detected in terms of self-reported dietary practices of the participants between baseline (Week 0), end of trial (Week 24), and end of discontinuation phase (Week 36). The majority (75.0%) of the participants on metformin endorsed having dieted in the past at baseline and none continually on a weight-loss diet through the end of trial and discontinuation phase; for the participants on placebo, the majority (66.7%) endorsed having dieted in the past at baseline and only one started on a continual diet to lose weight during the discontinuation phase. In terms of physical activity in the past seven days, participants on metformin reported increasing their time spent on doing moderate or vigorous activities across the three time points while decreasing their time sitting; however, participants on placebo reported decreasing their time spent on both doing moderate or vigorous physical activities and sitting.

## 4. Discussion

Our study helped shed light on the effects of metformin, a promising evidence-based pharmacological agent for the treatment of AIWG and metabolic dysfunction. There were direct clinical benefits to our study population. Participants in the metformin arm were able to control their weight better than the participants in the placebo arm throughout the 24 weeks of intervention. Despite the small sample size, there was a statistically significant difference between the metformin and placebo groups on weight outcomes. However, this difference between the two groups ceased in the 12 weeks after discontinuing the study drug. Metformin was also safe and tolerable for the participants, and its side effects were generally mild, tolerable, and resolved either spontaneously or with symptomatic treatment. It is also worth noting that side effects were reported by those on placebo as well.

The findings from our study are congruent with previous literature. A meta-analysis conducted by de Silva and colleagues [25] concluded that metformin compared to placebo resulted in a significant reduction in weight and BMI, but not in fasting blood sugar. Distinguishing our study from previous studies was our intention to enhance generalizability with a naturalistic approach, a longer trial duration of 24 weeks, and a 12-week discontinuation phase. A physical examination that was not part of the routine services was also included in the assessments as a precautionary measure. Despite the relatively long trial duration, the majority of the participants managed to complete at least 12 weeks of intervention. However, two participants dropped out from the trial, citing reasons such as perceived lack of study drug efficacy, from both the metformin and placebo arms. This suggests a discrepancy in expectations, as while the participant on metformin was not gaining as much weight as those on placebo, she was also not losing the significant amount of weight she had hoped for. This emphasizes the importance of educating the patients on the effects of metformin prior to use, and that for patients who have already gained a significant amount of weight, metformin may only be suitable and prescribed as an adjunctive weight control measure rather than a weight loss measure. 

A major limitation of this study was the small sample size and absence of a power calculation. Whilst the intention was for a pilot study to better understand the feasibility of conducting a future full-scale project, challenges were faced in recruiting participants who met the inclusion criteria and were willing to commit to the entire period of the clinical trial. The resulting small sample size limited the scale of inferential statistical tests that could be conducted. Hence, while the results from the mixed model analysis and RMANOVA were included for reference, they are not meant to be conclusive in nature and should be interpreted with caution. In addition, the small sample size also limited our ability to conclude if the effects of metformin could be sustained after discontinuation, considering that three out of five of the participants who completed the full intervention phase gained weight they previously lost during the discontinuation phase, one gained weight during intervention and lost weight during discontinuation, and one lost weight during intervention and continued losing weight during discontinuation.

The study included measures on diet and physical activity, in an attempt to ensure a well-rounded assessment of the participants’ physical health. However, this also gave rise to another limitation, due to the reliance of self-report methods alone for data collection. Previous literature had shown that self-report measures were prone to more variability and less reliability as compared to device measures [39,40]. Meanwhile, in this study, the self-reported diet and physical activity data showed that those on placebo perceived themselves to be putting in more effort to control or lose weight than those on metformin, albeit unsuccessfully. In addition, one of the participants on metformin reported an outlier value on the amount of time spent on moderate activities. As such, the self-reported diet and physical activity data should be interpreted with caution, and future studies with sufficient funds should include an objective measure or instrument to corroborate the self-reported data, such as a digital pedometer or a smartphone app.

## 5. Conclusions

To conclude, our results support the use of metformin as a safe and tolerable weight control measure in a typical outpatient sample of overweight young people with FEP. For more effective weight control, metformin should be prescribed as early as possible before significant weight changes occur, and preferably paired with behavioral or physical interventions on diet and activity [12,22]. Patients should also be educated on the effects of metformin, to mitigate the disappointment of not experiencing immediate weight loss and consequently dropping out or giving up. Results from this pilot study can be used to guide future more naturalistic research examining the use of metformin as part of treatment algorithms for management of AIWG, as well as study metformin in comparison to or combined with other strategies, such as behavioral interventions and antipsychotic switching. It is hoped that future studies will eventually help clinicians make relevant informed management decisions that maximize the clinical benefits while minimizing the adverse cardiometabolic consequences.

## Figures and Tables

**Table 1:** Schedule of procedures and assessments administered to participants at each study visit.

	Screening Phase	Intervention Phase					Discontinuation Phase	Early Termination
**Visit**	**1**	**2**	**3**	**4**	**5**	**6**	**7**	
**Week**	**−1**	**0**	**3**	**6**	**12**	**24**	**36**	
**Procedures/Assessments**								
Informed consent	🗸							
Sociodemographic and clinical characteristics	🗸							
Concomitant medication(s) review	🗸	🗸	🗸	🗸	🗸	🗸	🗸	🗸
Anthropometric measurements: Body weight (kg); height (m); body mass index (kg/m^2^); waist circumference (cm)	🗸	🗸	🗸	🗸	🗸	🗸	🗸	🗸
Vital signs: Systolic and diastolic blood pressure (mmHg); pulse rate (bpm)	🗸	🗸	🗸	🗸	🗸	🗸		🗸
Physical examination	🗸		🗸	🗸	🗸	🗸		🗸
12-lead electrocardiogram	🗸					🗸		🗸
Serum pregnancy test (for females of childbearing potential only)	🗸					🗸		🗸
Laboratory blood tests: Serum creatinine and estimated glomerular filtration rate; serum insulin; lactic acid level; liver function test; serum fasting glucose	🗸				🗸	🗸		🗸
Thyroid Function Test	🗸							
Hemoglobin A1c (HbA1c)	🗸				🗸	🗸		🗸
Total cholesterol, high-density and low-density lipoprotein, triglycerides	🗸				🗸	🗸		🗸
Clinician-rated questionnaires: BPRS; GAF		🗸				🗸	🗸	🗸
Participant-rated questionnaires: PHQ-9; DPQ; IPAQ		🗸				🗸	🗸	🗸
Randomization		🗸						
Study drug dispensed		🗸	🗸	🗸	🗸			
Adverse events monitoring			🗸	🗸	🗸	🗸		🗸
Drug accountability and adherence check			🗸	🗸	🗸	🗸		🗸

BPRS: Brief Psychiatric Rating Scale; GAF: Global Assessment of Functioning; PHQ-9: Patient Health Questionnaire 9-item version; DPQ: Dietary Preference Questionnaire; IPAQ: International Physical Activity Questionnaire.

**Table 2:** Baseline demographic and clinical characteristics of the enrolled participants (n = 17).

	Metformin (n = 8)	Placebo (n = 9)	*p*-Value
Age—years, mean (SD)	25.0 (3.9)	24.0 (6.0)	0.593
Gender—no. (%)			
- Male - Female	4 (50.0) 4 (50.0)	5 (55.6) 4 (44.4)	0.819
Ethnicity—no. (%)			
- Chinese - Malay - Indian - Others	4 (50.0) 1 (12.5) 1 (12.5) 2 (25.0)	6 (66.7) 2 (22.2) 1 (11.1) 0 (0.0)	0.443
Diagnosis—no. (%)			
- Schizophrenia - Schizophreniform disorder - Schizoaffective disorder - Psychotic disorder not otherwise specified - Mood disorders with psychotic features	5 (62.5) 1 (12.5) 2 (25.0) 0 (0.0) 0 (0.0)	4 (44.4) 1 (11.1) 1 (11.1) 1 (11.1) 2 (22.2)	0.494
Body weight—kg, mean (SD)	82.3 (17.1)	87.1 (11.5)	0.471
Height—m, mean (SD)	1.7 (0.1)	1.7 (0.1)	0.413
Body mass index—kg/m^2^, mean (SD)	27.9 (6.4)	30.7 (4.8)	0.229
Waist circumference—cm, mean (SD)	94.9 (11.2)	97.2 (9.1)	0.596
Systolic blood pressure—mmHg, mean (SD)	116.3 (11.3)	119.6 (13.6)	0.500
Diastolic blood pressure—mmHg, mean (SD)	73.9 (8.2)	63.2 (9.0)	0.030 *
Pulse rate—bpm, mean (SD)	78.9 (20.0)	86.7 (11.1)	0.163
Concurrent medications—no. (%)			
- Typical antipsychotics			
Flupentixol	1 (12.5)	2 (22.2)	
- Atypical antipsychotics			
Amisulpride Aripiprazole Clozapine Olanzapine Paliperidone Risperidone	1 (12.5) 1 (12.5) 0 (0.0) 0 (0.0) 2 (25.0) 3 (37.5)	0 (0.0) 2 (22.2) 1 (11.1) 1 (11.1) 1 (11.1) 3 (33.3)	-
- Anticholinergics - Antidepressants - Mood stabilizers - Benzodiazepines	1 (12.5) 2 (25.0) 2 (25.0) 0 (0.0)	0 (0.0) 3 (33.3) 1 (11.1) 1 (11.1)	

* *p* < 0.05.

**Table 3:** Clinical parameters of the enrolled participants over time, represented as the mean (SD).

	Visit 1 Week −1 (Metformin n = 8; Placebo n = 9)	Visit 2 Week 0 (Metformin n = 8; Placebo n = 9)	Visit 3 Week 3 (Metformin n = 7; Placebo n = 9)	Visit 4 Week 6 (Metformin n = 7; Placebo n = 9)	Visit 5 Week 12 (Metformin n = 7; Placebo n = 9)	Visit 6 Week 24 (Metformin n = 5; Placebo n = 8)	Visit 7 Week 36 (Metformin n = 5; Placebo n = 8)
Weight—kg Metformin Placebo	82.3 (17.1) 87.1 (11.5)	82.6 (17.3) 87.5 (11.7)	82.4 (18.9) 88.2 (11.7)	82.6 (18.9) 89.0 (11.5)	81.6 (19.5) 90.2 (12.0)	84.8 (21.6) 90.7 (13.9)	85.9 (22.2) 92.3 (13.3)
BMI—kg/m^2^ Metformin Placebo	27.9 (6.4) 30.7 (4.8)	28.2 (6.5) 30.9 (4.7)	27.6 (7.3) 31.2 (4.5)	27.6 (7.0) 31.5 (4.7)	27.3 (6.9) 31.8 (5.0)	28.7 (8.0) 31.0 (4.6)	28.9 (8.0) 31.7 (5.1)
Waist circumference—cm Metformin Placebo	94.9 (11.2) 97.2 (9.1)	95.5 (11.2) 99.6 (9.0)	96.6 (14.8) 99.6 (7.7)	93.3 (13.7) 101.0 (8.8)	95.3 (13.5) 100.7 (9.8)	95.6 (14.5) 101.1 (9.8)	96.4 (19.9) 98.8 (14.0)
Systolic BP—mmHg Metformin Placebo	116.3 (11.3) 119.6 (13.6)	111.9 (13.5) 120.1 (12.4)	117.7 (18.3) 117.4 (14.4)	116.7 (17.5) 120.0 (15.2)	119.7 (14.4) 116.4 (9.1)	112.2 (23.2) 121.6 (12.5)	-
Diastolic BP—mmHg Metformin Placebo	73.9 (8.2) 63.2 (9.0)	64.3 (7.8) 68.1 (9.6)	69.6 (12.1) 70.9 (9.5)	68.7 (10.3) 69.0 (11.5)	70.1 (10.5) 67.8 (7.3)	62.2 (11.4) 67.9 (6.6)	-
Pulse rate—bpm Metformin Placebo	78.9 (20.0) 86.7 (11.1)	82.0 (14.9) 90.3 (14.2)	76.9 (9.2) 93.1 (15.9)	77.0 (5.2) 89.6 (18.1)	82.1 (9.1) 88.0 (17.3)	75.2 (13.7) 82.4 (14.9)	-
BPRS total score Metformin Placebo	-	22.8 (4.1) 24.4 (3.5)	-	-	-	21.0 (2.6) 23.3 (4.5)	22.6 (4.7) 20.3 (3.8)
GAF score Metformin Placebo	-	71.5 (6.0) 70.7 (3.0)	-	-	-	76.5 (10.8) 77.4 (8.0)	75.2 (11.1) 81.4 (8.3)
PHQ-9 total score Metformin Placebo	-	2.9 (2.5) 6.4 (4.3)	-	-	-	3.8 (3.0) 5.0 (5.6)	3.8 (3.8) 6.3 (4.4)
Fasting glucose—mmol/L Metformin Placebo	4.6 (0.3) 4.7 (0.2)	-	-	-	4.6 (0.3) 4.9 (0.4)	4.5 (0.2) 5.0 (0.6)	-
Serum insulin—mU/L Metformin Placebo	11.0 (4.6) 16.6 (11.6)	-	-	-	17.8 (12.5) 17.6 (13.6)	10.3 (10.3) 24.3 (25.1)	-
Triglycerides—mmol/L Metformin Placebo	1.6 (1.0) 1.9 (1.0)	-	-	-	1.6 (1.0) 1.8 (1.0)	1.0 (0.4) 2.4 (1.7)	-
Total cholesterol—mmol/L Metformin Placebo	4.7 (0.7) 5.1 (0.7)	-	-	-	4.4 (1.0) 5.1 (1.2)	4.6 (1.4) 5.2 (1.2)	-
HDL cholesterol—mmol/L Metformin Placebo	1.1 (0.2) 1.2 (0.3)	-	-	-	1.1 (0.1) 1.1 (0.3)	1.2 (0.3) 1.0 (0.2)	-
LDL cholesterol—mmol/L Metformin Placebo	2.9 (0.6) 3.4 (0.9)	-	-	-	2.6 (0.6) 3.2 (0.9)	2.8 (1.2) 3.1 (0.8)	-
Serum creatinine—µmol/L Metformin Placebo	73.6 (14.4) 67.4 (14.1)	-	-	-	72.4 (11.2) 67.2 (12.1)	66.6 (12.0) 73.1 (13.0)	-
Lactic acid—mmol/L Metformin Placebo	1.5 (0.7) 1.7 (0.7)	-	-	-	1.4 (0.4) 1.5 (0.5)	1.4 (0.3) 1.5 (0.4)	-
AST—U/L Metformin Placebo	29.5 (16.5) 27.7 (7.2)	-	-	-	28.0 (12.8) 31.7 (13.8)	47.2 (51.6) 32.8 (16.1)	-
ALT—U/L Metformin Placebo	41.8 (38.0) 34.3 (19.4)	-	-	-	41.3 (24.9) 49.8 (50.5)	86.8 (127.1) 50.5 (58.0)	-
HbA1c—% Metformin Placebo	5.2 (0.5) 5.3 (0.3)	-	-	-	5.1 (0.2) 5.5 (0.6)	5.0 (0.4) 5.6 (0.7)	-

BMI: Body Mass Index; BP: blood pressure; BPRS: Brief Psychiatric Rating Scale; GAF: Global Assessment of Functioning; HDL: high-density lipoprotein; LDL: low-density lipoprotein; AST: aspartate transaminase; ALT: alanine transaminase; HbA1c: hemoglobin A1c.

**Table 4:** Results of the mixed model analysis and RMANOVAs.

Mixed Model Fixed Effects (Intervention Phase)	Unconditional Means Model: Model A	Unconditional Growth Model: Model B	Growth Model: Model C
Initial status—Intercept (Status error SE)	86.0 (3.6) **	84.8 (3.4) **	86.3 (4.8) **
Initial status—Intervention/control			−3.0 (7.0)
Rate of change—Intercept		0.4 (0.2)	0.8 (0.2) **
Rate of change—Intervention/control			−1.2 (0.5) **
Variance components			
- Level 1 Within person	3.6 (0.7) **	1.2 (0.3) **	1.2 (0.2) **
- Level 2 In initial status	216.3 (76.7) **	200.2 (71.2) **	210.0 (77.1) **
- Level 2 In rate of change		1.1 (0.5) **	0.7 (0.3) *
Pseudo R^2^ statistics			
- Level 1 Within person		0.7	
- Level 2 Rate of change			0.3
Goodness of fit			
- −2 Res Log Likelihood	411.1	378.7	366.2
- AIC	415.1	384.7	372.2
- BIC	416.7	387.2	374.7
**RMANOVA (Intervention phase)**			***F* value (*df*)**
Within Subjects			0.659 (1.8) ^a^
Between Groups			0.108 (1)
Group × Time Effect			4.780 (1.8) ^a^*
**RMANOVA (Discontinuation phase)**			***F*** **value (*df*)**
Within Subjects			3.974 (1)
Between Groups			0.407 (1)
Group × Time Effect			0.124 (1)

* *p* < 0.05; ** *p* < 0.01; ^a^ The original degree of freedom (*df*) for within subjects effect was 4. However, a significant Mauchly’s Test of Sphericity suggested a violation of the sphericity assumption. Hence, *F* test and *df* values based on the Greenhouse–Geisser corrections were used. AIC: Akaike Information Criterion; BIC: Bayesian Information Criterion.

## Data Availability

All the data from this study reside with the Office of Research, Institute of Mental Health. Data are not available for online access; however, readers who wish to gain access to the data can write to the Clinical Research Committee, Institute of Mental Health/Woodbridge Hospital Secretariat at IMHRESEARCH@imh.com.sg. Access can be granted subject to the Institutional Review Board (IRB) and the research collaborative agreement guidelines. This is a requirement mandated for this research study by our IRB and funders.
